# Handwriting processes when spelling morphologically complex words in children with and without Developmental Language Disorder

**DOI:** 10.3389/fpsyg.2023.1112462

**Published:** 2023-05-23

**Authors:** Sarah Critten, Vincent Connelly, Julie E. Dockrell, Ian R. Mundy, Lynsey O’Rourke, Laura Callaghan, Kirsty Walter

**Affiliations:** ^1^School of Education, Childhood, Youth and Sport, The Open University, Milton Keynes, United Kingdom; ^2^Department of Psychology, Health and Professional Development, Oxford Brookes University, Oxford, United Kingdom; ^3^Institute of Education, University College London, London, United Kingdom; ^4^School of Social Sciences, Birmingham City University, Birmingham, United Kingdom; ^5^Warwickshire Local Authority, Warwick, United Kingdom

**Keywords:** hand-writing, morphological spelling, Developmental Language Disorder, morphological decomposition effect, reading, children

## Abstract

**Introduction:**

Representations activated during handwriting production code information on morphological structure and reflect decomposition of the root and suffix. Children with Developmental Language Disorder (DLD) have significant difficulties in spelling morphologically complex words, but previous research has not sought evidence for a morphological decomposition effect via an examination of handwriting processes in this population.

**Method:**

Thirty-three children aged 9–10 years with DLD, 33 children matched for chronological age (CA), and 33 younger children aged 7–8 years matched for oral language ability (LA) completed a dictated spelling task (21 words; 12 with inflectional suffixes, nine with derivational suffixes). The task was completed on paper with an inking pen linked to a graphics tablet running the handwriting software Eye and Pen. Pause analyses and letter duration analyses were conducted.

**Results:**

The three groups showed similar handwriting processes, evidencing a morphological decomposition effect in a natural writing task. Pause durations observed at the root/suffix boundary were significantly longer than those occurring in the root. Letter durations were also significantly longer for the letter immediately prior to the boundary compared to the letter after it. Nevertheless, despite being commensurate to their LA matches for mean pause durations and letter durations, children with DLD were significantly poorer at spelling derivational morphemes. Handwriting processes did significantly predict spelling accuracy but to a much lesser extent compared to reading ability.

**Discussion:**

It is suggested that derivational spelling difficulties in DLD may derive more from problems with underspecified orthographic representations as opposed to handwriting processing differences.

## 1. Introduction

Children with Developmental Language Disorder (DLD) often have associated literacy difficulties including problems with spelling (Joye et al., 2019). Spelling morphologically complex words with bound morphemes comprising specific inflectional and derivational suffixes are especially challenging for this population (Windsor et al., 2000; Mackie and Dockrell, 2004; Silliman et al., 2006; Larkin et al., 2013; Critten et al., 2014). However, less is known about whether different morphological structures modulate the handwriting process in children with DLD, as they are known to do in adults and typically developing children (e.g., Quémart and Lambert, 2019).

The origin of morphological spelling difficulties in children with DLD may be due to both underspecified orthographic representations and slower handwriting. Indeed, problems with underlying orthographic representations have been observed in this population (e.g., McCarthy et al., 2012; Critten et al., 2014; Williams et al., 2021). If there are underspecified morphological-orthographic mappings of word roots versus suffixes, children with DLD may show less evidence of morphological decomposition in their writing processes and in turn, this could also contribute to a slowing of handwriting speed (Chenoweth and Hayes, 2003). Furthermore, there is evidence that slow handwriting may contribute to poorer spelling accuracy in children with DLD (Connelly et al., 2012) and that slow handwriting execution speed in typical children also influences spelling accuracy (Pontart et al., 2013). Alternatively, if children with DLD, do not show discernible handwriting problems in terms of the speed of their graphomotor processing, i.e., commensurate with their overall levels of language ability or no different to peers, then this indicates that their spelling difficulties are more likely to be representational in origin. The present study will explore the differential effects of handwriting processes and the quality of underlying representations on spelling by examining online, within-word handwriting processes in primary school-aged children with and without DLD while they attempt to spell morphologically complex words.

Developmental Language Disorder (DLD) denotes language difficulties of idiopathic origin and is characterized by significant difficulties in receptive and expressive language that are pervasive across the different components of language including vocabulary, phonology and grammatical structures (Carroll and Critten, 2020). Children with DLD tend to show a delay in their development of language apparent through usage of short, simple words and sentences, difficulties understanding instructions or following conversations, and grammatical errors which would be unusual for typically developing children (Carroll and Critten, 2020). This disorder has been known by different names, most recently as Specific Language Impairment, to which much of the previous literature discussed in this paper refers. However, we have adopted the current terminology and definition of DLD arising from the Delphi study of Bishop et al. (2017).

Recent meta-analytic findings show that children with DLD consistently show poorer spelling ability than their typically developing peers of the same age (Joye et al., 2019; Graham et al., 2020). Joye et al. also reported commensurate performance of children with DLD to younger children matched for language abilities suggesting a delay, rather than a difference, in their trajectory of spelling development (see also Cordewener et al., 2012). However, these meta-analytic findings were derived largely from measures of spelling accuracy at the word level (either dictated spelling tasks or within narrative/expository texts) and while the studies included in the analyses tested spellings of different word types, further analytic breakdown comparing performance of children with DLD to control groups on these different word types was not conducted. Therefore, further investigation employing a more fine-grained analytic approach to different measures of spelling accuracy and word type is merited if researchers are to fully understand the origin of spelling difficulties in DLD.

One potential area to focus this investigation on is the spelling of morphologically complex words. According to single studies, children aged 9–11 years with DLD are often unexpectedly poor (in comparison to language-matched controls) when spelling inflectional morphemes such as the regular past tense-ed, the regular plural-s (Windsor et al., 2000; Mackie and Dockrell, 2004; Silliman et al., 2006; Larkin et al., 2013) and derivational morphemes where there is a shift in phonology and/or orthography between the base and derived forms, e.g., please > pleas/ant (Critten et al., 2014). Qualitative differences in error type have also been reported where children with DLD are more likely to omit suffixes altogether and/or make phonologically implausible spelling attempts (e.g., Larkin et al., 2013; Critten et al., 2014). These studies demonstrate that an analytic approach that considers accuracy of both whole words and the bound morphemes in isolation can reveal subtle differences in spelling abilities between children with DLD and their language-matched peers that would not be anticipated compared to other findings (Cordewener et al., 2012; Joye et al., 2019).

Attempts to conceptualize why these morphemes are particularly challenging for children with DLD to spell have identified four potential underlying factors: general oral language ability (Bishop and Clarkson, 2003), morphological awareness (Windsor et al., 2000), phonological awareness (Dockrell and Connelly, 2013) and orthographic/reading ability (McCarthy et al., 2012). Critten et al. (2014) was the first study to examine all four factors together and concluded that underspecified or “fuzzy” underlying orthographic representations associated with poor phonological-orthographic mappings was the likely reason for these spelling difficulties (Snowling, 2000; Perfetti and Hart, 2002). Triple word form theory argues that accurate spelling is underpinned by high quality orthographic-phonological-morphological mappings (e.g., Daffern et al., 2015) but the exact influence of morphological processing (if any) in the spelling of children with DLD remained unclear. However, Critten et al.’s study, only considered the final product of the children’s spelling accuracy. It did not consider the nature of the writing process itself which may help reveal if morphological influences are evident and where any difficulties in underlying processes may be located.

There is an analytic approach to studying writing that combines offline methodology (e.g., a traditional writing/spelling task) with real-time analysis (e.g., the use of digitizing tablets) supported by handwriting software such as Eye and Pen (Alamargot et al., 2006) or MovAlyzeR (Neuroscript, 2018). This allows exploration of the dynamics of writing alongside the finished product (Lambert and Quémart, 2019). Two aspects of word writing can be considered simultaneously (Quémart and Lambert, 2019). First, spelling knowledge, i.e., the cognitive representations supporting the transcription of words into their orthographic form, as measured by dictated spelling tasks or copying tasks. Second, the graphomotor processes involved in letter writing itself, as measured by frequency and duration of pauses and individual letter durations within words.

The Cascade Model (Van Galen, 1991) posits that the spelling knowledge and graphomotor processes operate in a parallel, interactive, and co-operative fashion. Thus, it follows that difficulties in the cognitive aspects of spelling can have a negative effect on handwriting performance (Lambert and Quémart, 2019). Indeed, a study of text-level handwriting processes in children with DLD indicated that spelling difficulties appeared to interrupt the flow of children’s transcription and were associated with shorter bursts of writing (Connelly et al., 2012). The converse effect can also be observed in typically developing children whereby inefficiencies in the graphomotor process may interfere with access to spelling representations, especially in younger writers where there is more dysfluency (Pontart et al., 2013). Examining word-level writing processes has the potential to help distinguish different sources of difficulty affecting the spelling of bound morphemes in children with DLD.

Letter duration analyses have been utilized to explore the online processes underlying word writing. The level (phonemic, morphemic, syllabic) and number of linguistic units activated in spelling a word have been shown to have an impact on real time handwriting processes when writing the word. Examinations of latencies when spelling morphologically complex words have revealed a morphological decomposition effect whereby written letter durations become increasingly longer as the boundary between the root and suffix approaches but shorten again after the boundary (Kandel et al., 2012). This finding suggests that parallel representations activated during handwriting production reflect information coded about morphological structure and so contributes to the decomposition of the root and suffix during the time course of handwriting. As the processing of the suffix occurs in parallel with the writing of the root, the cognitive load increases and so handwriting slows down. Once the handwriting of the root is completed then the cognitive load decreases and so handwriting speeds up again for the production of the suffix. Indeed, highly controlled copying tasks with French-speaking adults have shown that inter-letter interval durations between roots and suffixes are longer in suffixed compared to pseudo-suffixed words (Kandel et al., 2008) and letter durations of the last letter of the root, immediately prior to the suffix, were longer in suffixed compared to pseudo-suffixed words (Kandel et al., 2012). Pseudo-suffixed words have the same letters at the end of the word as their suffixed match, e.g., geolette versus boulette but-ette does not serve as a suffix in goelette- (Kandel et al., 2008). These findings (Kandel et al., 2008, 2012) support the idea that activation of representations during handwriting does not occur in a linear, letter-by-letter fashion but instead reflects the organization of underlying linguistic typology such as morpheme boundaries which in turn modulates the handwriting process (Lambert and Quémart, 2019).

To our knowledge, only one study has examined the effect of morphological structure in children’s within-word handwriting. Quémart and Lambert (2019) gave copying tasks to children aged 9–12 years and found that the older children (11–12 years) did show a decomposition effect as evidenced by longer durations for the letters preceding the morphemic boundaries compared to post boundary. The findings of Quémart and Lambert came from French-speaking children and therefore it is unknown at what age English-speaking children would show a morphological decomposition effect given orthographic differences between languages. Indeed, many studies of spelling accuracy in English-speaking children aged 10 years and younger do show evidence for morphological processing (e.g., Treiman and Cassar, 1996; Deacon, 2008; Breadmore and Carroll, 2016) suggesting a morphological decomposition effect may manifest earlier in development compared to French-speaking children. However, given the difficulties children with DLD have in spelling morphologically complex words, and derivational suffixes in particular, an examination of whether these children would show a morphological decomposition effect equivalent to their typically developing peers is merited.

In addition to these conceptual questions, there are also two methodological issues to consider. First the Kandel et al. (2008, 2012) and Quémart and Lambert (2019) studies employed copying tasks rather than traditional spelling-to-dictation tasks. Copying is different from spelling in that visual word input is involved and therefore the effects of morphological structure in copying could be due to processes involved in reading, processes involved in spelling, or some kind of interaction between them (Breadmore and Deacon, 2019). Indeed, written latencies are longer in copying tasks compared to spelling dictation tasks for children with spelling difficulties and are attributed to problems with reading accuracy (Afonso et al., 2020). In that sense a spelling dictation task is a more direct measure of the cognitive processes of spelling as it requires auditory analysis and spoken word recognition but not visual word recognition (Breadmore and Deacon, 2019). The second issue is that participants in the previous studies were asked to complete copying tasks in discursive uppercase letters. This contrasts to spelling to dictation tasks given in a school setting where children typically write in lowercase letters and use a mixture of discursive and cursive writing dependent on age and ability. It is important to consider the ecological validity of writing tasks used by researchers with young children (Franken and Harris, 2021). Therefore, it is currently unknown whether the effects of morphological structure would be apparent in a more naturalistic spelling task.

It has been established that children with DLD struggle when spelling most word types compared to peer groups of the same age although their achievement is generally in-line with their overall language abilities (Joye et al., 2019; Graham et al., 2020). In contrast, studies specifically examining the spelling of morphologically complex words have suggested that children with DLD may have additional difficulties when spelling some inflectional and derivational suffixes (e.g., Larkin et al., 2013; Critten et al., 2014) and this could be due to underspecified underlying orthographic representations (McCarthy et al., 2012; Critten et al., 2014). However, as these previous spelling studies of children with DLD only examined writing products (i.e., spelling accuracy) the first aim of the present study is to also examine online handwriting processes in an attempt to capture evidence of morphological decomposition in English-speaking children with DLD and their typically developing peers.

In order to do so, children completed the spelling task on a graphics tablet and letter and pause durations were extracted using handwriting software. This will be the first study (to our knowledge) to utilize this methodology in English-speaking children with and without DLD and will explore whether morphological representations modulate the handwriting process in these groups of children as they have been shown to do in French speakers. The online handwriting processes will be examined in two ways. First, letter durations will be compared for the letters immediately before and after the boundary between the word roots and suffixes. Second, a pause analysis will be conducted to measure mean pause durations in the word roots compared to those at the boundaries between the word roots and suffixes.

The second aim is to examine the predictions made by the Cascade Model (Van Galen, 1991). Exploration of word-level writing processes in children with and without DLD has the potential to confirm the parallel and interactive operation of spelling knowledge and graphomotor processes. Slow and effortful handwriting may contribute to a larger cognitive load during spelling and lead to poorer spelling accuracy in children with and without DLD (Connelly et al., 2012; Pontart et al., 2013). In addition, exploration of children’s spelling knowledge and writing processes, alongside other, related skills can elucidate whether spelling accuracy is mainly due to cognitive factors such as the quality of orthographic representations, phonological and morphological awareness, and oral language ability or whether the fluency of graphomotor processes also make an additional, unique contribution to spelling attainment. In contrast to previous online spelling studies (e.g., Kandel et al., 2008, 2012; Quémart and Lambert, 2019) a naturalistic spelling-to-dictation spelling task will be used rather than a copying task (Breadmore and Deacon, 2019).

The following research questions will be addressed:

1. Will there be any differences in word and bound morpheme spelling accuracy (product), mean pause durations (process), and mean letter production durations (process) according to language ability (DLD/CA/LA) and word type (inflectional/derivational)?

It is predicted that children with DLD will be poorer than their chronological age matches but commensurate to their language age matches in their word spelling (e.g., Joye et al., 2019) but that children with DLD will also be poorer at spelling inflectional and derivational morphemes than their language age matches (e.g., Larkin et al., 2013; Critten et al., 2014). Following Quémart and Lambert (2019) it is also predicted that the chronological age matched children may show a morphological decomposition effect in their letter and pause durations. It was not anticipated that this effect would also be found in children with DLD (due to their problems with spelling morphologically complex words) or their language age matches (as they were much younger than the Quémart and Lambert sample). Therefore, the duration of the letter at the end of the root would not be significantly longer than the duration of the letter at the start of the suffix. Furthermore, pause length at the boundary would not be significantly longer than pauses in the root.

2. Will there be a relationship between handwriting processes (mean pause and letter durations) and spelling accuracy?

It is predicted that there will be negative correlations between spelling accuracy and the pause and letter durations whereby as accuracy increases, durations decrease, thus supporting the notion that effortful handwriting may constrain the spelling process (Van Galen, 1991; Connelly et al., 2012; Pontart et al., 2013).

3. Will handwriting processes account for any unique variance in spelling accuracy after controlling for other cognitive, language, and literacy variables?

It is predicted that reading (McCarthy et al., 2012; Critten et al., 2014) and handwriting processes (Van Galen, 1991; Connelly et al., 2012; Pontart et al., 2013) will significantly predict spelling accuracy.

## 2. Method

### 2.1. Design and participants

The participants in this study were recruited as part of a wider project examining the relationship between oral and written language in children with DLD. Findings have previously been reported relating to text-level writing abilities (Connelly et al., 2012) and morphological spelling abilities (Critten et al., 2014). The standardized scores for children’s general cognitive, language and literacy abilities (Tables 1, 2) have been reported previously (Critten et al., 2014). However, the spelling accuracy and handwriting analyses of this experimental spelling task (conducted during a three-month longitudinal follow-up) are unique to the current study and previously unreported.

**Table 1:** Means, (standard deviations), f score, df, *p-*value, effect size and Bonferroni *post hoc* results (where applicable) for screening measures per group: DLD, CA, LA.

Measure	DLD	CA	LA	*F*	df	*p*	Partial η^2^	Bonferroni *post hoc*
Core language standard score (CELF)	68.45 (5.53)	102.88 (11.34)	93.61 (7.92)					
Nonverbal abilities: matrices ability score (BAS)	96.06 (6.52)	104.96 (9.68)	98.96 (7.84)					
Matching variables								
Age in years/months (SD in months)	9/10 (3.6)	9/10 (2.9)	8/1 (6.2)	244.30	2.96	<0.001	0.84	DLD=CA > LA
Formulated sentences raw score (CELF)	31.42 (4.19)	47.52 (4.44)	31.25 (4.23)	155.81	2.96	<0.001	0.77	DLD = LA, SLI < CA LA < CA
Spelling raw score (BAS)	16.33 (4.31)	22.31 (5.25)	16.64 (4.85)	16.08	2.96	<0.001	0.25	DLD = LA, SLI < CA LA < CA
Spelling ability score (BAS)	85.00 (16.47)	121.12 (16.35)	94.42 (13.34)	48.50	2.96	<0.001	0.50	DLD < LA < CA

Table taken from Critten et al. (2014).

**Table 2:** Means, standard deviations, f score, df, p value, effect size and Bonferroni *post hoc* results for language and literacy measures per group: SLI, CA, LA.

Measure	DLD	CA	LA	*F*	df	*p*	Partial η^2^	Bonferroni *post hoc*
Inflectional morphological awareness raw score	10.94 (1.39)	12.85 (0.36)	12.06 (0.97)	30.37	2,96	<0.001	0.39	DLD < LA < CA
Inflectional morphological awareness z score	−0.79 (1.09)	0.71 (0.29)	0.09 (0.76)	30.37	2,96	<0.001	0.39	DLD < LA < CA
Derivational morphological awareness raw score	5.12 (1.11)	5.79 (0.48)	5.30 (0.95)	4.95	2,96	0.009	0.09	DLD < CA DLD = LA CA = LA
Derivational morphological awareness z score	−0.31 (1.20)	0.41 (0.52)	−0.11 (1.02)	4.95	2,96	0.009	0.09	DLD < CA DLD = LA DLD = LA
Phonological elision raw score (CTOPP)	10.78 (4.28)	17.18 (2.91)	14.18 (4.18)	22.91	2,96	<0.001	0.32	DLD < LA < CA
Phonological rhyme raw score (PhAB)	12.27 (4.49)	18.36 (3.10)	17.06 (3.41)	24.58	2,96	<0.001	0.34	DLD < CA DLD < LA CA = LA
Phonological awareness z score (elision z + rhyme z)	−1.51 (1.58)	1.22 (1.05)	0.29 (1.27)	36.49	2,96	<0.001	0.43	DLD < LA < CA
Single word reading raw score (YARC)	31.61 (11.05)	49.18 (5.85)	38.72 (8.02)	35.02	2,96	<0.001	0.42	DLD < LA < CA
Single word reading z score (YARC)	−0.73 (0.99)	0.84 (0.52)	−0.09 (0.72)	35.02	2,96	<0.001	0.42	DLD < LA < CA

Table taken from Critten et al. (2014).

Ninety-nine children were assigned to one of three matched groups. There was a total of 33 children with DLD (22 boys mean age = 9:10 years, *SD* = 3.57 months, range = 11 months). A further 33 children were matched for chronological age (CA) and gender (mean age = 9:10 years, *SD* = 2.94 months, range = 10 months) and another 33 children (LA) were matched for gender, language, and single word spelling abilities (mean age = 8;10 years, *SD* = 6.25 months, range = 7 months). All children had English as their first language and were predominantly of white, British ethnicity. Social Economic Status (SES) was controlled across schools by confirming that the percentage of children receiving free school meals (a strong indicator of SES in the UK) was in the average range, although this was not controlled for statistically in our analyses.

To recruit a sample of children with DLD, professionals, across five counties in southern England, were asked to nominate children who had specific language impairments (but no diagnosed or discernible handwriting or motor difficulties). Participants were screened to confirm diagnosis using the four core sub-tests of the Clinical Evaluation of Language Fundamentals, 4th edition (CELF-4 UK, Semel et al., 2006): concepts and following directions, recalling sentences, formulated sentences, word classes (receptive and expressive). For a diagnosis of DLD, children had to achieve a standard composite score of 75 or below (2 SDs below the mean). The matrices test from the British Ability Scales, 2nd Edition (BAS II: Elliott et al., 1997) established nonverbal abilities within the average range. As Table 1 shows all participants met the criteria for DLD according to typical diagnostic practice at the time, with a significant difference between their CELF-4 test score and their BAS II matrices test: *t*(64) = 15.39, *p* < 0.001, *r* = 0.89.

The two groups of comparison children attended the same primary schools as those diagnosed with DLD, and were selected by teachers on the basis of average attainment on curriculum assessments and no additional learning needs. The CA comparison children were confirmed as having language ability and nonverbal ability within the average range using the same CELF-4 UK core tests and the BAS II matrices. The children were matched in age to the children with DLD within 3 months and the groups did not differ in mean age.

The LA comparison children also had scores on language and nonverbal ability within the average range and were matched with the children with DLD using their raw score on the formulated sentences task from the CELF-4 UK. The LA comparison children were also matched to the DLD group using their raw score on the single word spelling task from the BAS II.

### 2.2. Measures

As previously mentioned, screening for the DLD and control groups was confirmed using the CELF-4 UK (Semel et al., 2006) and the BAS II Matrices subtest (Elliott et al., 1997) to establish general oral language ability and nonverbal ability, respectively. Further standardized tests were used to measure spelling ability (BAS II Spelling subtest, Elliott et al., 1997), Phonological awareness (the elision test from the Complete Test Of Phonological Processing: CTOPP; Wagner et al., 1999 and the rhyme task from the Phonological Assessment Battery: PhAB; Frederickson et al., 1997), Inflectional and derivational morphological awareness: (derived from selected items on the CELF-4 UK Word Structure task, Semel et al., 2006) and reading (Single Word Reading Task from the York Assessment of Reading Comprehension: YARC, Snowling et al., 2009; for full details of the tasks and reliability/validity information please see Critten et al., 2014).

#### 2.2.1. Experimental morphological spelling task

A list of 21 words (derived from Critten et al., 2014) was presented as a dictated spelling test, delivered in a randomized order. There were 12 words containing inflectional morphemes; 6 regular past tense verbs containing-ed, e.g., *killed* and 6 regular plural nouns, e.g., *houses*. There were nine words containing derivational morphemes: three where there was a phonological shift from the root word to the derived form, e.g., *confidence*, three where there was an orthographic shift from the root word to the derived form, e.g., *easily* and three where there were both phonological and orthographic shifts, e.g., *severity*.

Written word frequency was checked using the UK derived Children’s Printed Word Database (Masterson et al., 2003). This demonstrated that the frequency of the inflectional words ranged from 3 to 498 and that the derivational words were generally less frequent, as would be expected, ranging from 3 to 330. See Appendix 1 for the complete word list and written word frequency scores.

### 2.3. Procedure

All children were assessed individually in a quiet room at school. Ethical approval for the study had been gained in line with guidelines from the British Psychological Society (BPS) through Oxford Brookes University ethics committee and informed consent from schools, parents and children was provided prior to any testing. During the screening process the CELF core tests, BAS matrices and BAS spelling were administered in two testing sessions. The phonological awareness and morphological awareness tasks were delivered over a third testing session. Finally, the experimental morphological spelling test was delivered in a fourth testing session, 3 months later. Children were allowed to terminate the sessions if they wished. However, no child terminated the sessions since the organization of data collection into different sessions resulted in manageable time periods of testing for the children.

All standardized tests were administered according to the procedures in the manual. The morphological spelling task was completed by children on A4 lined paper taped to a digitizing tablet (100 Hz, Intuos 4; Wacom, Vancouver, Washington) and recorded by Eye and Pen handwriting software (Version 1; University of Poitiers, Poitiers, France). Children were given an inking pen to write their spellings with. The tablet surface records the xy coordinates of the pen’s position to a Windows based computer (Alamargot et al., 2006). The procedure is therefore identical for the child to a typical written composition task undertaken in the classroom. The researcher verbally presented each word to be spelled in isolation, in the context of a sentence and then in isolation again and children were asked to write out the word. Children were given no further instructions about how they should write their spellings thus enabling naturalistic handwriting samples.

#### 2.3.1. Handwriting analysis

##### 2.3.1.1. Pauses

Careful consideration was given to the threshold chosen to measure the within-word pauses in our data. Generally speaking, pause duration has been examined at text-level for handwriting studies in an attempt to capture writers in flow versus when they are pausing due to increased cognitive effort, e.g., retrieving a spelling, planning, or revising. These are often thought of as higher order “cognitive” processing pauses (Alamargot et al., 2020). Among these studies there has been considerable variation in the thresholds used from 130 ms to 5 s (Olive et al., 2009), although the majority adopt around 2 s (e.g., Connelly et al., 2012). However, there are far fewer studies that have examined within word handwriting pauses.

One that has (Alamargot et al., 2020) defines the process of handwriting as not only comprising the action of moving the pen to form the letter strokes but also pausing (either with the pen down on the paper or pen in the air movements) in order to control the movements being made, e.g., the location and size of the strokes. These handwriting pauses are likely to be shorter than 130 ms, occur more frequently and are located both within and between letters. Alamargot et al. gave children with Dyslexia aged 11 years and two control groups, one matched for chronological age and one matched for orthographic skills (spelling and grammar) two writing tasks and examined their pausing behaviors and durations. They concluded that shorter within word handwriting process pauses (as opposed to higher order “cognitive” pauses) could be classified as those lasting 20–199 ms.

Given that the aim of the present study was to look at within word handwriting processes, it seemed appropriate therefore to adopt a threshold of 20 ms as this is the lower end of Alamargot et al.’s classification. Incidentally this was also the minimum threshold that could be used in Eye and Pen (Alamargot et al., 2006). Using this low threshold ensured a thorough and fine-grained analysis of handwriting movements. Pauses were identified when the pen nib was not moving on the page for at least 20 ms, i.e., in the air or in contact with the page but still. Pauses that occurred at the morphological boundary, between the root word and the suffix, were classified as “boundary” pauses. Pauses that occurred within the root of the word were classified as “root” pauses. Any pauses that occurred after the boundary were not included in analysis. The length of each pause was recorded in milliseconds.

The position of each morphological boundary was established using those identified in the English Lexicon Project.^1^ The ELP affords access to a large set of English words (40,481) with identified lexical characteristics, including morphology.

All spelling attempts were included in the analyses irrespective of accuracy with the exception of misspellings that did not seem to include a morphological boundary, see later explanation. At the word level, there were 2079 spelling attempts of which 1,061 were correct (51% accuracy). Of these there were 1,188 inflection attempts of which 701 were correct (59% accuracy) and 891 derivation attempts of which 360 were correct (40% accuracy). At the suffix level, 71% of words contained a correctly spelled suffix. Of these 84% of inflection attempts contained correctly spelled inflectional morphemes while 54% contained correctly spelled derivational morphemes.

For misspellings, the position of the morphological boundary was decided by the research team using a systematic approach described below. On the rare occasion that disagreements occurred, the lead author instigated a discussion and resolutions were agreed 100% of the time. In misspellings where the suffix was correctly spelled but the root word had been spelled incorrectly, it was decided that the boundary lies before the suffix. For example, in the word *drawers*, the addition of the suffix “*s*” turns the root word (*drawer*) into a plural, therefore, the boundary is between the “*r*” and “*s*” (*drawer-s*). When *drawers* was misspelled, e.g., *darwas*, it was judged that the “*s*” was used correctly to create a plural, thus the boundary was between the second “*a*” and the “*s*,” (*darwa-s*).

Some misspellings did not include a correctly spelled suffix, but the suffix could be identified from the phonology of the word. For example, in the word *learned*, “*ed*” is the suffix which creates the past-tense verb, therefore the morphological boundary is between the “*n*” and the “*e*” (*learn-ed*). When *learned* was misspelled as *lerand,* it was decided that the addition of the “*d*” provides the same sound as adding “*ed*” and was an attempt to create the past tense of the root, and so the boundary lies between the “*n*” and the “*d*” *(leran-d).*

For misspellings which did not have an obvious boundary, the lead author used her experience of spelling error analysis and working with children with spelling difficulties to identify the boundary between root and suffix. For example, *sevante* (*sever-ity*) was divided as *sevan-te*. On the rare occasion that misspellings did not contain a morphological boundary, for example, if a child did not clearly attempt both parts of the word, such as *opne* for (*open-ed*), *feild* (*field-s*), or *covtn* (*confidence*) the words were not included in the data.

**Figure 1:**
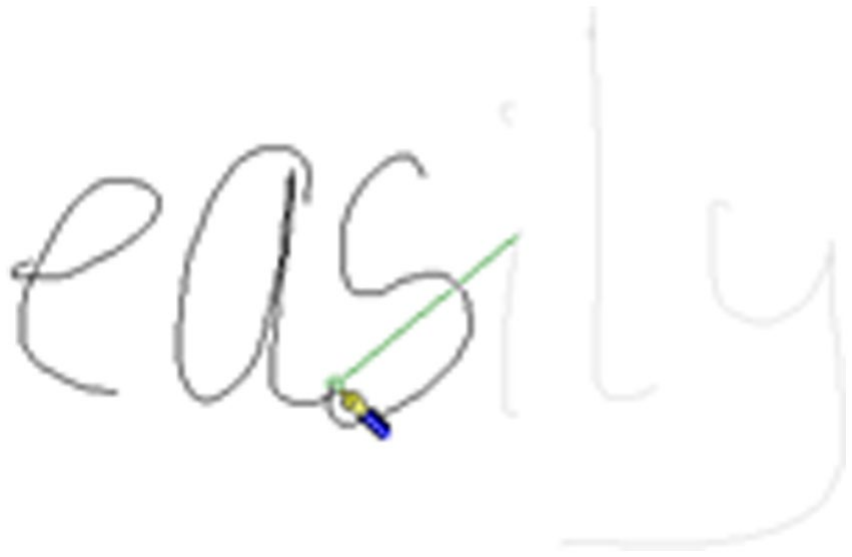
Handwriting example A from a child for the word *easily*.

**Figure 2:**
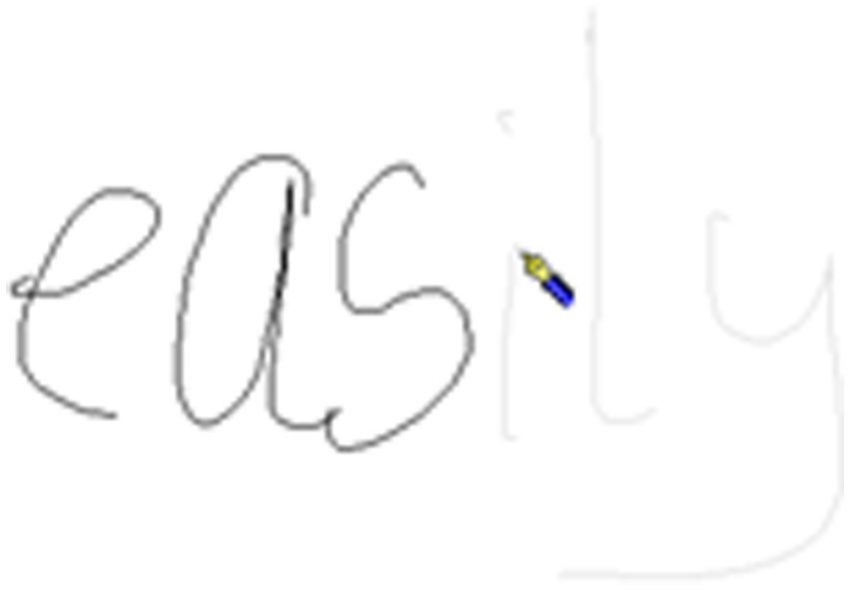
Handwriting example B from a child for the word *easily*. Note for Figure 1 and this figure: In Figure 1, the pen icon indicates the final moment where the pen was in contact with the page during the production of the letter “*s*.” The green line indicates where the pen travels to. In this figure the pen icon indicates the first contact the pen has with the page for production of the letter “*i*.” The time between the final contact of pen on paper for the letter “*s*” and the first contact for the letter “*i*” is the morphological boundary. In this case, there is a single pause of 92 ms at the morphological boundary.

Children had been instructed to write as they normally would and therefore sometimes used cursive (joined) handwriting, and sometimes left spaces between the letters (discursive). When letters were clearly separated with a space, morphological boundaries were from the final pen-up of the letter before the boundary to the first pen-down of the letter after the boundary, see Figures 1, 2 for a handwritten example of the word *easily* with a pen-up between letters.

**Figure 3:**
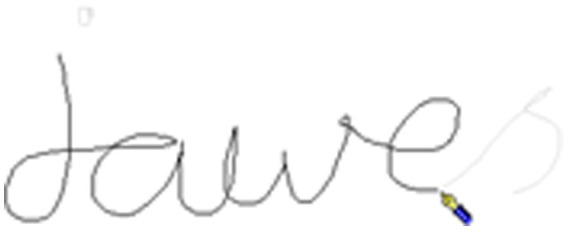
Handwriting example A from a child for the world *jaws*.

**Figure 4:**
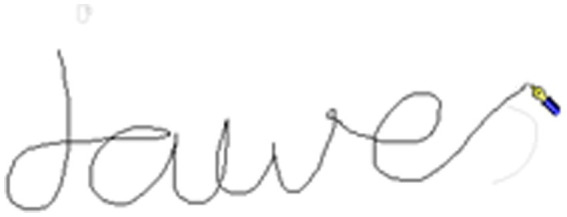
Handwriting example B from a child for the world *jaws*. Note for Figure 3 and this figure: The morphological boundary is the stroke that joins the “*e*” and “*s*” (jawe-s). Figure 3 shows the pen icon at the start of the joining stroke. This figure shows the pen icon at the end of the joining stroke. There were 9 boundary pauses over 20 ms with a sum of 320 ms during this joining stroke.

When the letters either side of the boundary were joined (in cursive script) the boundary included linking strokes. For example, Figures 3, 4 shows the word *jaws*, written as *jawes* with the morphological boundary between the “*e*” and the “*s*” (jawe-s), with the “*e*” and “*s*” joined. As illustrated by Figure 3, the stroke joining the letters either side of the morphological boundary starts at the change of pen trajectory at the end of the “*e*” and ends at the change in pen trajectory at the start of the “*s*” (end of stroke shown in Figure 4).

##### 2.3.1.2. Boundary letter duration

The time taken to produce each letter positioned immediately before or after the boundary was measured in milliseconds; for example, in the word *severity* (*sever-ity*) both the “*r*” and “*i*” were measured. When the pen was lifted off the page between letters, the letter duration was measured from the initial pen-down of the letter to the final pen-lift of that letter.

Letters that were joined (in cursive script) were separated by the change of pen trajectory that occurred between letters as one letter is completed and the next letter starts (based on a procedure by Kandel and Perret, 2015). The joining stroke is included as part of the letter that it leads into rather than the preceding letter. In many words, the post boundary letter was also the final letter of the word. Therefore, including the joining stroke preceding the letter, as opposed to the joining stroke after the letter, should minimize the overall impact of joining strokes on length of letter duration.

Pauses within letter production were included in the time taken to produce the letter. Including intra-letter pauses provides an overall picture of time taken to produce each letter, as opposed to measuring the individual components of letter writing such as the speed of the pen movements or the time taken to produce individual letter strokes.

## 3. Results

### 3.1. Data preparation

A pause was defined as a break in fluent writing lasting for 20 ms or more. Analyses of the mean pause durations are presented below followed by analyses of children’s production durations for letters immediately prior to and following the root/morpheme boundary. All statistical analyses were conducted in JASP, version 0.9 (JASP Team, 2018).

### 3.2. Spelling accuracy

Children’s word-level and morpheme-level spelling accuracy is summarized by language group in Table 3. One-way ANOVA revealed significant group differences in inflectional [whole words: *F*(2, 91) = 18.30, *p* < 0.001; morphemes: *F*(2, 91) = 15.90, *p* < 0.001], derivational [whole words: *F*(2, 91) = 34.25, *p* < 0.001; morphemes: *F*(2, 91) = 28.21, *p* < 0.001], and overall spelling accuracy [whole words: *F*(2, 91) = 28.80, *p* < 0.001; morphemes: *F*(2, 91) = 29.01, *p* < 0.001].

**Table 3:** Mean [95% CI] spelling accuracy by language group.

	Word-level accuracy		
	DLD	CA	LA
Inflections (max. = 12)	5.17 [4.06, 6.27]	9.21 [8.34, 10.09]	6.55 [5.56, 7.54]
Derivations (max. = 9)	2.03 [1.32, 2.75]	5.76 [5.06, 6.46]	3.06 [2.46, 3.67]
Total (max. =21)	7.20 [5.50, 8.90]	14.97 [13.59, 16.35]	9.61 [8.11, 11.11]
	Morpheme accuracy		
	DLD	CA	LA
Inflections (max. = 12)	8.87 [7.93, 9.81]	11.48 [11.15, 11.82]	9.97 [9.29, 10.64]
Derivations (max. = 9)	3.03 [2.13, 3.93]	6.85 [6.26, 7.44]	4.48 [3.75, 5.22]
Total (max. =21)	11.90 [10.25, 13.55]	18.33 [17.57, 19.09]	14.45 [13.24, 15.66]

Bonferroni corrected pairwise comparisons revealed that children with DLD performed significantly below the level of the CA controls for inflectional (whole words: *p* < 0.001, *d* = 1.49; morphemes: *p* < 0.001, *d* = 1.41), derivational (whole words: *p* < 0.001, *d* = 1.91; morphemes: *p* < 0.001, *d* = 1.86), and overall spelling accuracy (whole words: *p* < 0.001, *d* = 1.84; morphemes: *p* < 0.001, *d* = 1.88). Word-level spelling accuracy of children with DLD was not significantly different from their LA controls, nor did performance differ for inflectional morpheme spelling accuracy (all *p*s > 0.07 when controlling for multiple comparisons), although effect sizes were moderate (*d* = 0.50), However, the DLD children were significantly impaired in relation to the LA controls in their spelling of derivational morphemes (*p* = 0.020, *d* = 0.66) and for overall morpheme spelling accuracy (*p* = 0.012, *d* = 0.66). The CA controls were also significantly more accurate than the LA controls for inflections (whole words: *p* < 0.001, *d* = 1.03; morphemes: *p* = 0.005, *d* = 1.05), derivations (whole words: *p* < 0.001, d = 1.48; morphemes: *p* < 0.001, *d* = 1.29), and overall (whole words: *p* < 0.001, *d* = 1.34; morphemes: *p* < 0.001, *d* = 1.40).

### 3.3. Mean pause duration

Due to equipment failure no pause data were obtained for three of the children (one with DLD and two LA controls). In addition, two further children with DLD were excluded because they registered extremely long pauses in multiple conditions, even in comparison to other DLD children. Therefore, a final sample of 94 children (33 CA controls, 31 LA controls, and 30 with DLD) were included in the pause analyses.

A 3 (language group: DLD, CA control, LA control) by 2 (part of word: root, boundary) by 2 (word type: inflection, derivation) mixed factorial ANOVA was conducted with mean pause duration as the dependent variable. The analysis revealed a significant effect of language group [*F*(2, 91) = 6.33, *p* = 0.003, *η_p_*^2^ = 0.122]. *Post hoc* analyses with Bonferroni corrections applied to control for multiple comparisons revealed that the average pause length for children with DLD was approximately 100 ms longer than for CA controls (*M* difference = 97.52, *p* = 0.002, *d* = 0.366). The average pause length among children with DLD was not significantly different to that observed among LA controls (*M* difference = 44.98, *p* = 0.331). The difference in mean pause duration between the CA and LA control groups was also non-significant (*M* difference = 52.54, *p* = 0.171). The ANOVA analysis also confirmed a significant main effect for part of word [*F*(1, 91) = 45.99, *p* < 0.001, *η_p_*^2^ = 0.336], with the average pause length being significantly longer at the boundary than in the root. On average, children paused 10.73 times, 95% CI [9.87, 11.59], when producing word roots (reflecting the multiple letters they comprise) and 1.65 times, 95% CI [1.15, 2.15], at word boundaries (as these are only between the last letter of the root and the first letter of the suffix). When combined with the pause duration data this demonstrates that children were showing large numbers of relatively brief pauses while producing word roots. In contrast, at morphological boundaries, children’s pauses are less numerous but also substantially longer.

There were two significant interaction effects in the mean pause duration data. The first of these was between language group and part of word [*F*(2, 91) = 3.90, *p* = 0.024, *η_p_*^2^ = 0.079], indicating that the group differences in mean pause duration were more pronounced at the boundary than within the root. The second interaction was between word type and part of word [*F*(1, 91) = 11.64, *p* < 0.001, *η_p_*^2^ = 0.113] and reflected the fact that differences in mean duration between boundary pauses and root pauses were more pronounced for inflections than derivations. The three-way interaction was non-significant (*p* = 0.092), as was the interaction between language group and word type (*p* = 0.386). The main effect of word type was also non-significant (*p* = 0.085).

### 3.4. Boundary letter duration

A further six children (five CA controls and one LA control) were excluded from the boundary letter duration analysis after equipment failure meant that boundary letter duration data were not recorded for these children. Therefore, these analyses were conducted with a sample of 88 children (28 CA controls, 30 LA controls, and 30 with DLD).

A 3 (language group: DLD, CA control, LA control) by 2 (letter position: pre-, post-boundary) by 2 (word type: inflection, derivation) mixed factorial ANOVA was conducted with mean boundary letter duration as the dependent variable. The analysis revealed a significant effect of language group [*F*(2, 85) = 6.39, *p* = 0.003, *η_p_*^2^ = 0.131]. *Post hoc* analyses with Bonferroni corrections applied to control for multiple comparisons revealed that the average boundary letter duration for children with DLD was significantly longer than for CA controls (*M* difference = 191.18, *p* = 0.009, *d* = 0.33), but did not differ significantly to that observed among LA controls (*M* difference = 7.26, *p* > 0.999). The LA controls also had significantly longer boundary letter durations than the CA controls (*M* difference = 198.45, *p* = 0.006, *d* = 0.34). The ANOVA analysis also confirmed a significant main effect for letter position [*F*(1, 85) = 9.28, *p* < 0.003, *η_p_*^2^ = 0.098], with the average boundary letter duration being significantly longer pre-boundary compared to post-boundary (*M* difference = 51.09, *p* = 0.003, *d* = 0.32). Despite the trend for boundary letter durations to be marginally longer in inflections than in derivations, the main effect of word type was non-significant (*M* difference = 35.61, *p* = 0.079), as were the interaction effects (range of *p*-values: 0.199–0.761).

### 3.5. Predicting spelling accuracy

Further analyses were conducted to investigate the associations between children’s pause and letter durations, word-level spelling accuracy, oral language ability (raw scores from the CELF Formulating Sentences subscale), morphological awareness (raw scores from the CELF Word Structure subscales), phonological awareness (summed z-scores from the CTOPP Phoneme Elision and PhAB Rhyme subscales), and word reading (raw scores from YARC Single-word Reading subscale). For these analyses, the children’s pause durations and boundary letter durations were converted to z-scores and summed to create a composite variable (known henceforth as “writing composite”) capturing both sources of individual differences in writing behavior during the spelling task. Separate analyses were conducted for inflections and derivations. An additional child from the LA control group had to be excluded from some of these analyses due to them missing data from one of the CELF assessments, giving a final sample of 87 children (28 CA controls, 29 LA controls, and 30 with DLD).

Partial correlations were calculated between the variables in each group, controlling for non-verbal IQ (raw scores from the BAS Matrices subscale), with a focus on children’s processing of inflections (Table 4) and derivations (Table 5). Of primary interest were the correlations between the writing composite, spelling accuracy, and the other measures of language and literacy ability.

**Table 4:** Partial correlations between the writing composite, spelling accuracy, and other language and literacy measures in each group (inflections).

	1	2	3	4	5	6
DLD						
1. Spelling accuracy (inflections)	–	−0.076	0.548**	−0.126	0.735***	−0.465*
2. Oral language ability		–	−0.119	−0.132	0.077	−0.108
3. Phonological awareness			–	−0.303	0.759***	−0.353
4. Morphological awareness (inflections)				–	−0.047	0.260
5. Word reading					–	−0.403
6. Writing composite (inflections)						–
CA control						
1. Spelling accuracy (inflections)	–	0.137	0.555**	0.279	0.773***	−0.204
2. Oral language ability		–	0.131	−0.063	0.189	0.000
3. Phonological awareness			–	0.121	0.509**	−0.194
4. Morphological awareness (inflections)				–	0.260	−0.436*
5. Word reading					–	−0.280
6. Writing composite (inflections)						–
LA control						
1. Spelling accuracy (inflections)	–	0.049	0.450*	0.018	0.708***	−0.613**
2. Oral language ability		–	0.413*	0.151	0.076	0.040
3. Phonological awareness			–	0.009	0.629***	−0.044
4. Morphological awareness (inflections)				–	−0.060	−0.297
5. Word reading					–	−0.463*
6. Writing composite (inflections)						–

***p < 0.001, **p < 0.01, *p < 0.05.

**Table 5:** Partial correlations between the writing composite, spelling accuracy, and other language and literacy measures in each group (derivations).

	1	2	3	4	5	6
DLD						
1. Spelling accuracy (derivations)	–	−0.090	0.684***	0.046	0.835***	−0.466*
2. Oral language ability		–	−0.119	−0.120	0.077	0.151
3. Phonological awareness			–	0.039	0.759***	−0.380*
4. Morphological awareness (derivations)				–	0.013	0.090
5. Word reading					–	−0.431*
6. Writing composite (derivations)						–
CA control						
1. Spelling accuracy (derivations)	–	0.349	0.453*	0.438*	0.777***	−0.391
2. Oral language ability		–	0.131	0.281	0.189	0.052
3. Phonological awareness			–	0.389*	0.509**	−0.261
4. Morphological awareness (derivations)				–	0.524**	−0.343
5. Word reading					–	−0.371
6. Writing composite (derivations)						–
LA control						
1. Spelling accuracy (derivations)	–	0.066	0.366	−0.092	0.693**	−0.542**
2. Oral language ability		–	0.413*	0.261	0.076	−0.100
3. Phonological awareness			–	0.264	0.629***	−0.029
4. Morphological awareness (derivations)				–	0.025	0.252
5. Word reading					–	−0.331
6. Writing composite (derivations)						–

***p < 0.001, **p < 0.01, *p < 0.05.

There were broad similarities in the pattern of correlations obtained in the three groups of children across the different word types, as well as some subtle differences. For example, significant, negative correlations were observed between the writing composite and spelling accuracy (of both word types) for the children with DLD and the LA controls, indicating that children with longer pause durations and lower accuracy scores on the spelling test were associated, but this relationship was weaker and non-significant in the CA control group. The same pattern was also observed for the correlation between the writing composite and single-word reading, with significant, negative relationships observed in children with DLD. However, while the LA controls showed this same pattern for the inflections, they were much more similar to the CA controls for the derivations with a notably weaker relationship. There was also a correlation (marginal for inflections) between the writing composite and phonological awareness for children with DLD, but the same association was not observed for the CA or LA controls. Finally, while a significant, negative correlation between morphological awareness and the writing composite was observed for the CA controls, this relationship was weaker and statistically non-significant in the LA control group and these variables were positively related (although non-significantly) for children with DLD.

Finally, hierarchical regression analyses were conducted to explore whether the writing composite could account for any additional variance in spelling accuracy over and above that explained by the other literacy, language and background measures. Data were collapsed across language groups for these analyses, in order to maximize statistical power. Age, non-verbal IQ, oral language, morphological awareness, phonological awareness and single-word reading were all entered at the first-step, with the composite variable reflecting children’s pause and letter durations added as an additional predictor at step two. As previously, separate analyses were conducted for inflections and derivations and the first analysis focused on children’s processing of inflections. The initial model accounted approximately 64% of the variance in children’s inflectional spelling accuracy [*F*(6, 80) = 23.68, *p* < 0.001, *R*^2^ = 0.640]. Single-word reading was found to be a significant predictor of spelling accuracy (*β* = 0.769, *p* < 0.001). There were no other significant predictors in this model (*p* > 0.25 in all cases). When added to the model at step two, the writing composite was found to account for an additional 2.6% of the variance in inflectional spelling accuracy [Δ*R*^2^ = 0.026, *F*(1, 79) = 6.17, *p* = 0.015]. Furthermore, it was a significant predictor of spelling accuracy within the expanded model (*β* = −0.199, *p* = 0.015), along with single-word reading (*β* = 0.650, *p* < 0.001). There were no other significant predictors (*p* > 0.30 in all cases).

A similar pattern of results emerged in the analysis of derivational spelling. The initial model accounted for approximately 73% of the variance in children’s derivational spelling accuracy [*F*(6, 80) = 37.89, *p* < 0.001, *R*^2^ = 0.737]. Single-word reading was again found to be a significant predictor of spelling accuracy (*β* = 0.751, *p* < 0.001) and oral language ability also approached significance (*β* = 0.148, *p* < 0.076). There were no other significant predictors in the initial model (*p* > 0.20 in all cases). When added to the model at step two, the writing composite was found to account for an additional 2.2% of the variance in derivational spelling accuracy [Δ*R*^2^ = 0.022, *F*(1, 79) = 7.39, *p* = 0.008]. The writing composite was again a significant predictor of spelling accuracy within the expanded model (*β* = −0.175, *p* = 0.008), along with single-word reading (*β* = 0.664, *p* < 0.001), and a marginal effect of oral language ability (*β* = 0.146, *p* < 0.070). There were no other significant predictors (*p* > 0.30 in all cases).

## 4. Discussion

The present study had three research questions in examining online handwriting processes when spelling morphologically complex words in children with DLD compared to chronological and language-aged, matched controls. First to compare spelling accuracy (product), pause durations (process) and letter durations (process) according to group of children (DLD, CA, LA), word type (inflectional, derivational) and letter position (pre-boundary, post boundary). Second to examine relationships between spelling accuracy, pause durations and boundary letter durations. Third to look at predictors of spelling accuracy.

In line with the meta-analytic findings of Joye et al. (2019), children with DLD, while poorer at spelling whole words compared to their CA matches, were commensurate in ability to their younger LA matches. This demonstrated that they were achieving as expected for their language ability. However, the more fine-grained analysis revealed that while the inflectional suffix accuracy of children with DLD was also commensurate with their LA matches (contrary to Larkin et al., 2013) they were significantly poorer when spelling derivational suffixes compared to both control groups thus confirming the difficulties shown by the sample of children with DLD in Critten et al. (2014) remained consistent overtime.

Revealingly, a morphological decomposition effect was observed for all three groups of children in both the boundary letter duration analyses (letter durations were longer for the final letter of the root, immediately prior to the morphemic boundary, compared to the first letter of the inflectional/derivational suffix) and pause analyses (root pauses were shorter and more frequent while boundary pauses were fewer and significantly longer). This replicates the findings of French-speaking adults (Kandel et al., 2008, 2012) and children aged 11–12 years (Quémart and Lambert, 2019) and extends them by evidencing morphological decomposition in the context of a more naturalistic spelling dictation task (Breadmore and Deacon, 2019) and in younger English-speaking children aged 7–10 years. These data also add to the existing spelling literature evidencing morphological processing in English-speaking children from a young age (e.g., Treiman and Cassar, 1996; Deacon, 2008; Breadmore and Carroll, 2016). More importantly this finding confirmed that children with DLD showed the same modulation of handwriting processes influenced by underlying morphological structure as both control groups, despite the fact that their handwriting was generally slower and their spelling less accurate than chronological age-matched peers. This suggested that differences in online handwriting processes could be ruled out as the origin of reduced spelling accuracy for derivational suffixes in children with DLD.

Correlational analyses confirmed the predictions of Van Galen’s handwriting model in that a close relationship was shown between spelling knowledge and graphomotor processes (as measured by our combined handwriting process variable). The children with DLD were slow handwrites compared to their CA matches but performed equivalently to their LA peers. Effortful graphomotor processes could negatively impact upon access to spelling representations perhaps by placing an additional strain on cognitive resources. However, while regression analyses did indicate that handwriting processes made a unique contribution to both inflectional and derivational spelling accuracy, it was negligible compared to the contribution made by reading ability.

Our sample of children with DLD were poorer at single word reading compared to both control groups (Table 2) suggesting underlying orthographic representations that are underspecified in terms of orthographic-phonological mappings (Snowling, 2000; Perfetti and Hart, 2002). Thus difficulties in the cognitive aspects of spelling, could have a negative effect on handwriting performance (Lambert and Quémart, 2019). This has also been shown previously in text-level analyses of writing performance in children with DLD (Connelly et al., 2012). Despite this, children with DLD did show a morphological decomposition effect building upon previous findings (e.g., Critten et al., 2014) to demonstrate a morphological influence within their spelling as triple word form theory predicts (Daffern et al., 2015). However, while this (perhaps) implicit processing effect of morphological structuring is evident, children with DLD are still slower at learning the letter patterns for certain derivational suffixes than would be expected for their language abilities.

### 4.1. Limitations and future directions

The findings relating to derivational morpheme spelling accuracy could be viewed as unexpected (e.g., Joye et al., 2019). However, the meta-analytic studies of Joye et al. (2019) and Graham et al. (2020) expose two key areas of concern in the spelling literature of DLD. First the lack of studies that also include an LA control group as well as a CA group and second, the lack of studies that have specifically examined derivational spelling accuracy. This supports the need for replication studies in different samples of children with DLD.

A further relevant consideration arises from the derivational spelling difficulties evidenced in Critten et al. (2014) and that the present study is a single sample effect since the same children participated in both studies, albeit at different points in time. Alternatively, it is arguable that our use of more fine-grained analyses of different linguistic units within words has simply uncovered an aspect of spelling that children with DLD find particularly challenging. A further connected point is that we only tested the children’s spelling accuracy for the derived forms rather than conducting separate tests of the roots and derived forms in isolation. A comparison of these in future may also be useful in elucidating our data on derivational spelling in DLD.

Another key aspect to consider is our use of handwriting analyses in a fairly uncontrolled dictated spelling task. We have argued this is as an advantage as it is ecologically valid and reflective of how children complete spelling tests in schools. However, it cannot be ignored that previous studies controlled for variables such as word length and the identity of letters occurring around root/morpheme boundaries. Despite this limitation, the present study has replicated the previous findings derived from this controlled methodology (Kandel et al., 2008, 2012; Quémart and Lambert, 2019) thus confirming the robustness of the morphological decomposition effect.

By not controlling for word length, another consideration is that the increase in pause duration at the boundaries compared to the roots could be due to a general slowing effect rather than actually reflecting any underlying morphological organization. However, the letter duration findings would contradict this idea of a general slowing effect as letter durations after the boundary were shorter than the letter durations immediately prior to it. Future studies could rule out this idea of a slowing effect more definitively by giving a naturalistic spelling dictation task but also controlling for word length.

The data analyzed in the present study included many spelling errors and this is in contrast to previous studies that controlled for spelling errors by using copying tasks to ensure accuracy (e.g., Quémart and Lambert, 2019). Consequently, we had to make judgments within the research team about how best to interpret these errors. Although inter-rater reliability checks were very high, arguably there is still margin for error that could have impacted our results and how they were interpreted. We are confident this type of risk is merited and mitigated by the advantages of employing a more naturalistic spelling dictation task. This is a purer and more ecologically valid representation of the spelling process and the errors that children make (Breadmore and Deacon, 2019). Furthermore, copying latencies are known to be longer for children with spelling difficulties (Afonso et al., 2020) due to interaction with reading accuracy. We were keen to avoid that potential confound particularly as Critten et al. (2014) found that reading is the main predictor of morphological spelling ability. Finally, and as mentioned earlier our data have replicated the previous findings of a morphological decomposition effect across all three groups with varying amounts of errors adding further weight to the validity of including misspellings in the data set.

A further issue to consider is the pause threshold of 20 ms adopted in this study. The aim of this study was to examine within word handwriting processes. However, when looking at handwriting studies that examined pause duration it became apparent that the term “pause” has often been applied arbitrarily to different types of pausing behavior. Higher order “cognitive” pauses are often examined at text level and are longer (approximately 2 s) and less frequent compared to within word pauses that are much shorter (e.g., 20–199 ms), more frequent and occur within and between letters (Olive et al., 2009; Alamargot et al., 2020). Our study has captured all forms of within word pausing behavior but future research could be more discerning and perhaps compare within letter versus between letter pauses and compare the outcomes from a range of thresholds. Our data ranges show that children from the CA group (the most efficient hand-writers) had minimum values of 40 ms for root pauses and 30 ms for boundary pauses. This difference of only 10–20 ms to Alamargot et al.’s recommendation that within word pauses start from 20 ms, lends support to their representativeness for this age group of children.

A final consideration for future studies is the nature of the DLD sample in the present study. They had no reported or discernible handwriting or motor difficulties. However, given the level of co-occurrence between DLD and Developmental Coordination Disorder (Lino and Chieffo, 2022), a study that also had a DLD + DCD group may indicate a greater influence for graphomotor processes when spelling morphologically complex words and uncover further differences from language matched controls. Furthermore, this sample could be construed as quite intellectually able given they were matched for non-verbal abilities with the CA controls. A study including children with a range of intellectual abilities may again, uncover different findings.

### 4.2. Implications

Theoretically speaking these findings support suggestions that orthographic representations are coded or organized according to linguistic structures such as morphemes and provide evidence that this can be seen in samples of natural writing obtained from young children aged 7–10 years with and without DLD. These findings have also clarified understanding of why children with DLD struggle to spell morphologically complex words, suggesting the content of the orthographic representations themselves rather than differences in online processing seem likely to be the key reason for their difficulties with spelling accuracy. From an educational perspective, the evidence of the morphological organization/structuring within their spelling suggests a delay rather than a difference in the morphological spelling ability of children with DLD. However specific difficulties with derivational suffixes suggest spelling instruction targeted at these letter patterns would be beneficial.

## Data availability statement

The raw data supporting the conclusions of this article will be made available by the authors, without undue reservation.

## Ethics statement

The studies involving human participants were reviewed and approved by the Oxford Brookes Ethics Committee, Oxford Brookes University. Written informed consent to participate in this study was provided by the participants’ legal guardian/next of kin.

## Author contributions

SC, VC, JD, and KW contributed to the conception and design on the study. SC and KW collected and coded the standardized data and spelling data. LO’R and LC prepared the pause and letter duration data. IM analyzed the data and wrote the Results section. SC wrote the Introduction, Method, and Discussion sections. All authors contributed to manuscript revision, read, and approved the submitted version.

## Funding

This research was supported by funding from the Leverhulme Trust and the Economic and Social Research Council (ESRC).

## Conflict of interest

The authors declare that the research was conducted in the absence of any commercial or financial relationships that could be construed as a potential conflict of interest.

## Publisher’s note

All claims expressed in this article are solely those of the authors and do not necessarily represent those of their affiliated organizations, or those of the publisher, the editors and the reviewers. Any product that may be evaluated in this article, or claim that may be made by its manufacturer, is not guaranteed or endorsed by the publisher.

## Footnotes

^1^ https://elexicon.wustl.edu/index.html

## Appendix 1

Words used in the spelling task according to inflectional (regular past tense verbs, regular plural nouns) and derivational (phonological shift, orthographic shift, phonological and orthographic shift) morpheme type.

**Table:**

Inflectional: Regular past tense verbs	Inflectional: Regular plural nouns	Derivational phonological shift	Derivational orthographic shift	Derivational: phonological and orthographic shift
Killed (30)	Houses (333)	Magician (3)	Hungry (330)	Natural (11)
Opened (333)	Eyes (498)	Discussion (3)	Scary (32)	Severity (3)
Started (398)	Fields (92)	Convertible (3)	Easily (54)	Confidence (3)
Stopped (454)	Fees (3)			
Missed (51)	Drawers (8)			
Learned (41)	Jaws (35)			

Written word frequency (Masterson et al., 2003) is presented in parentheses.
